# Antioxidant Activity In Vitro and Protective Effects Against Lipopolysaccharide-Induced Oxidative Stress and Inflammation in RAW264.7 Cells of *Ulva prolifera*-Derived Bioactive Peptides Identified by Virtual Screening, Molecular Docking, and Dynamics Simulations

**DOI:** 10.3390/foods14132202

**Published:** 2025-06-23

**Authors:** Jiasi Liu, Zhiyong Li, Huiyue Gu, Songdong Shen

**Affiliations:** 1 School of Basic Medical Sciences, Soochow University, Suzhou 215101, China; 20224221018@stu.suda.edu.cn (J.L.); 20224221011@stu.suda.edu.cn (H.G.); 2 Suzhou Chien-Shiung Institute of Technology, Suzhou 215101, China; 0178@csit.edu.cn

**Keywords:** *Ulva prolifera*, bioactive peptides, virtual screening, Keap1-Nrf2 pathway, oxidative stress

## Abstract

Large-scale blooms of *Ulva prolifera* severely impact coastal ecosystems and economic development. In addressing Ulva management, the development of high-value utilization approaches for this macroalga remains crucial. Compared to other marine algae, *Ulva prolifera* exhibits higher protein content with diverse amino acid profiles, and existing studies demonstrate that hydrolyzed *Ulva prolifera* proteins can yield biologically active peptides with functional potential. Conventional methods for producing bioactive peptides are often cost-intensive. Here, we employed in silico enzymatic hydrolysis to generate small peptides from *Ulva prolifera* protein. Through computer screening, molecular docking with the Keap1 protein, and molecular dynamics simulations, we identified a potential antioxidant peptide, DWS (Asp-Trp-Ser). Molecular docking and dynamics simulations revealed that DWS forms stable complexes with Keap1 by establishing hydrogen bonds and Pi bonds with conserved amino acid residues (Leu557, Gly558, Ile559, Val604, Val606, and Arg415). In vitro antioxidant assays demonstrated that DWS exhibits potent DPPH and ABTS radical scavenging activities as well as reducing power. Cellular experiments showed that DWS effectively alleviates LPS-induced oxidative stress and inflammation in RAW264.7 macrophages.

## 1. Introduction

Under normal physiological conditions, cellular metabolism generates reactive oxygen species (ROS), which accumulate in cells and not only do not cause cellular damage, but also have a specific biological function [1]. When an organism is exposed to external stimulation, there is an imbalance between the accumulation of ROS and the endogenous antioxidant system of the cells, which in consequence causes oxidative stress (OS) [2]. Under such conditions, excessive ROS oxidatively modify proteins, lipids, and nucleic acids, triggering inflammatory responses and, in severe cases, necrotic cell death [3,4]. The development of a variety of diseases, such as atherosclerosis, chronic obstructive pulmonary disease, type 2 diabetes mellitus, and various cardiovascular diseases, is often associated with oxidative stress [5].

Numerous studies have shown that targeting the Kelch-like ECH-associated protein 1 (Keap1)–nuclear factor erythroid 2-related factor 2 (Nrf2)–antioxidant response element (ARE) signaling pathway may play a protective role in several oxidative-stress- and inflammation-related diseases [6]. Keap1 is a negative regulator of Nrf2. Under normal conditions, Keap1 binds to the ETGE sequence of nrf2 through its Kelch active region and mediates the ubiquitination degradation of Nrf2 [7]. The disruption of the interaction between Keap1 and Nrf2 has been demonstrated to be a contributing mechanism to the release and translocation of Nrf2 into the nucleus, thereby activating downstream antioxidant response elements [8]. Therefore, the development of bioactive compounds that competitively bind keap1 with nrf2 is an effective strategy to improve the body’s resistance to oxidative stress, and bioactive peptides are a promising candidate for this purpose. Bioactive peptides are defined as short amino acid sequences, typically consisting of 2 to 20 amino acid residues, with a molecular weight of under 6 kDa [9]. Bioactive peptides can be derived from diverse food-derived proteins. While intrinsically inactive within their original protein sequences, these peptides are liberated via enzymatic hydrolysis, microbial fermentation, or gastrointestinal digestion, subsequently exhibiting specific physiological functions [10]. Food-derived bioactive peptides have garnered increasing research attention due to their green and healthy nature, safety and non-toxicity, and easy absorption. Numerous studies have been conducted to prepare active peptides with antioxidant and anti-inflammatory benefits from food-derived proteins [11,12,13,14].

With advances in bioinformatics, computer-aided virtual screening is gaining prominence in bioactive peptide research owing to its speed and cost-effectiveness, including the virtual digestion of protein sequences, the prediction of potential biological activities, molecular docking, and molecular dynamics (MD) simulations [15,16,17]. Molecular docking is a computational approach used to study interactions between small molecules (ligands) and biomacromolecules (receptors), analyze plausible conformations, and predict binding modes along with their binding affinities [18]. An MD simulation is an atomistic computational method that models molecular motions based on classical mechanics [19]. MD simulations are commonly employed to investigate the conformational dynamics and structural stability of ligand–receptor complexes, providing validation of molecular docking predictions [20]. Many researchers have identified diverse antioxidant compounds, including bioactive peptides, through molecular docking and MD simulations targeting Keap1 [21,22,23,24].

*Ulva prolifera* is a marine green macroalga widely distributed along China’s coastal regions, particularly in Fujian Province and the Yellow–Bohai Sea area [25]. In recent years, increased eutrophication of marine water and deterioration of the climate have led to the massive growth of *Ulva prolifera*, which in turn triggers green tides, seriously affecting maritime transport as well as offshore ecosystems and safety [26,27]. However, it is worth emphasizing that *Ulva prolifera* is an algae resource with considerable utilization value. Studies have shown that *Ulva prolifera* possesses substantial nutritional value, characterized by high protein content and a complete amino acid profile including most essential amino acids required by humans [28,29]. Numerous studies have demonstrated that marine algal proteins possess antioxidant and anti-inflammatory properties, highlighting their potential for development as functional food ingredients [30,31]. Furthermore, research has shown that hydrolyzed *Ulva prolifera* proteins can yield biologically active peptides with specific functional activities [32]. Therefore, the development of bioactive peptides from *Ulva prolifera* represents a promising approach for its high-value utilization. This study aimed to screen *Ulva prolifera* small-molecule peptides with potential antioxidant activity through computer-aided virtual screening, combined with molecular docking and MD simulations, and integrated computational approaches, including in silico enzymatic hydrolysis, virtual screening, molecular docking, and molecular dynamics simulations. The selected peptides were chemically synthesized, and their protective effects on inflammation and oxidative stress were investigated in lipopolysaccharide (LPS)-stimulated RAW264.7 macrophages.

## 2. Materials and Methods

### 2.1. Materials

*Ulva prolifera* protein sequences from the National Center for Biotechnology Information (NCBI) (https://www.ncbi.nlm.nih.gov/, accessed on 10 April 2023) are shown in Table S1. DPPH (1,1-Diphenyl-2-picrylhydrazyl) and ABTS (2,2′-azinobis-(3-ethylbenzothiazoline-6-sulfonic acid) were purchased from Aladdin Biochemical Technology, (Shanghai, China). MTT (Methylthiazolyldiphenyl-tetrazolium bromide), a Reactive Oxygen Species Assay Kit, a Total Superoxide Dismutase Assay Kit with NBT, and a Nitric Oxide Assay Kit were purchased from Beyotime (Shanghai, China). A Catalase (CAT) assay kit (Visible light) and a Malondialdehyde (MDA) assay kit (TBA method) were purchased from Nanjing Jiancheng Bioengineering (Nanjing, China). Hifair^®^ AdvanceFast 1st Strand cDNA Synthesis SuperMix for qPCR and Hieff UNICON^®^ Universal Blue qPCR SYBR Green Master Mix were purchased from Yeasen Biotechnology (Shanghai, China). The RAW264.7 cell line and cell culture medium were purchased from Haixing Biotechnology (Suzhou, China). All other reagents were of analytical reagent grade.

### 2.2. Computer-Aided Virtual Screening

The *Ulva prolifera* proteins screened above were virtually digested using the online tool PeptideCutter (https://web.expasy.org/peptide_cutter/, accessed on 10 April 2023) [33], selecting peptides containing 2-6 amino acid residues and removing repetitive sequences. The potential biological activity of the above-selected peptides was predicted using PeptideRanker (http://distilldeep.ucd.ie/PeptideRanker/, accessed on 11 April 2023) [34], selecting peptides with a score of >0.5. Using Proteomics tools (http://www.innovagen.com/proteomics-tools, accessed on 11 April 2023), the solubility of the above-selected peptides was predicted, and peptides with good water solubility were selected. The reported peptide sequences were removed by screening through the online database BIOPEP-UWM (https://biochemia.uwm.edu.pl/biopep-uwm/, accessed on 12 April 2023) [35]. The ADMET properties of the peptides were predicted using the online tool admetSAR (https://lmmd.ecust.edu.cn/admetsar1/predict/, accessed on 12 April 2023) [36], and the metrics selected for this experiment mainly included Caco-2 cell permeability, human intestinal absorption (HIA), blood–brain barrier penetration (BBB), metabolic substrate or inhibitor, and toxicity.

### 2.3. Molecular Docking

Molecular docking was performed using the Discovery Studio 2019 Client program to investigate the interaction between the above-screened peptides and the Keap1 protein. Ligand preparation proceeded as follows: based on the sequences of the screened peptides, we selected different amino acids; constructed 3D structures of small-molecule ligands; and performed ionization, optimization of isomeric states, and energy minimization of the ligand models. Receptor preparation proceeded as follows: we downloaded the Keap1 protein (ID: 4XMB) from the PDB database (https://www.rcsb.org/, accessed on 13 April 2023) [37], saved it in PDB format, removed the original ligands and water molecules from the complex, hydrogenated the receptor, optimized the conformation, and created the active site cavity. The active site of Keap1 was defined at coordinates X: −3.71, Y: 0.71, Z: −17.51. Semi-flexible docking between Keap1 and bioactive peptides was performed using the CDOCKER module with the CHARMm force field, while retaining default parameters for other settings. The molecular docking of Keap1 (PDB ID: 4XMB) with its native ligand (2,2′-(naphthalene-1,4-diylbis(((4-methoxyphenyl)sulfonyl)azanediyl))diacetamide) was performed as a control.

### 2.4. MD Simulations

The protein–ligand complex obtained from molecular docking was subjected to molecular dynamics simulation using GROMACS 2020.3. The AMBER99SB-ILDN force field and the general AMBER force field (GAFF) were employed to generate the parameters and topology files for the protein and ligand, respectively. The simulation box dimensions were set to ensure a minimum distance of 1.0 nm between any protein atom and the box boundary. The system was solvated with SPC216 water molecules, and water molecules were replaced with Na^+^ and Cl^−^ ions to neutralize the system. Energy minimization was performed using the steepest descent algorithm to eliminate unfavorable atomic contacts or steric clashes. To ensure proper equilibration, 100 ps NVT and NPT ensemble simulations were conducted at 300 K and 1 bar, respectively. Subsequently, a 50 ns molecular dynamics (MD) simulation was performed under periodic boundary conditions, with temperature (300 K) and pressure (1 bar) controlled by the V-rescale and Parrinello–Rahman methods, respectively. The integration time step was set to 2 fs. Long-range electrostatic interactions were calculated using the Particle Mesh Ewald (PME) method with a Fourier spacing of 0.16 nm, while all bond lengths were constrained using the LINCS algorithm. Trajectory visualization, analysis, and animation were performed using VMD 1.9.3 and PyMOL 2.4.1.

### 2.5. Synthesis of Peptides

Selected peptides with the highest potential antioxidant activity were synthesized by Shanghai Hongtai Biotechnology with a purity greater than 95%.

### 2.6. In Vitro Antioxidant Activity Assay

The in vitro antioxidant capacity of peptides was determined using DPPH radical scavenging, ABTS radical scavenging, and reducing capacity.

The DPPH radical scavenging capacity was determined according to the method of Liu et al. [38] with minor modifications. First, 0.5 mL of different concentrations of the sample or vitamin C solutions was added to a 1.5 mL microcentrifuge tube, and for the blank control group, the same volume of ddH_2_O was used instead of the sample solution, and then 0.5 mL of DPPH ethanol solution was added, followed by thorough mixing. The plate was incubated for 30 min at room temperature under dark conditions, and the absorbance values of the groups were determined at 517 nm by an ultraviolet–visible spectrophotometer. The scavenging capacity of DPPH radicals was calculated as follows:

$$\mathrm{DPPH}\;\mathrm{radical}\;\mathrm{scavenging}\;\mathrm{activity}\;(100\%)=\left(1-\frac{A_{1}}{A_{2}}\right)\times 100\%$$

where A_1_ represents the absorbance of the sample and vitamin C groups and A_2_ represents the absorbance of the blank control group.

The ABTS radical scavenging capacity was determined according to the method of Rei et al. [39] with minor modifications. First, 20 μL of different concentrations of samples or vitamin C solution was added to a 96-well plate, while 20 μL of PBS solution was added to the blank control group, and 200 μL of ABTS working solution was added to each well and mixed thoroughly, followed by thorough mixing. The plate was incubated for 30 min at room temperature under dark conditions, and the absorbance values of the groups were determined at 517 nm by an ultraviolet–visible spectrophotometer. The scavenging capacity of ABTS radicals was calculated as follows:

$$\mathrm{ABTS}\;\mathrm{radical}\;\mathrm{scavenging}\;\mathrm{activity}\;(100\%)=\left(1-\frac{A_{1}}{A_{2}}\right)\times 100\%$$

where A_1_ represents the absorbance of the sample and vitamin C groups and A_2_ represents the absorbance of the blank control group.

The reducing capacity was determined according to the method of Thaipong et al. [40] with minor modifications. In a 10 mL microcentrifuge tube, 0.25 mL of sample solution or vitamin C solution was mixed with 1.25 mL of PBS (pH 7.4), followed by 1.25 mL of 1% (*w*/*v*) K_3_[Fe(CN)_6_]. The mixture was incubated at 45 °C for 15 min. Subsequently, 1.25 mL of 10% (*w*/*v*) TCA was added, and the solution was vortexed thoroughly. After centrifugation at 3000 rpm for 10 min, 1.25 mL of the supernatant was transferred to a 5 mL tube and mixed with 2.5 mL of ddH_2_O and 0.25 mL of 0.1% (*w*/*v*) FeCl_3_. The reaction proceeded at room temperature for 15 min, and the absorbance values of the groups were determined at 700 nm by an ultraviolet–visible spectrophotometer. Higher absorbance values indicate greater reducing capacity.

### 2.7. Cell Culture of RAW264.7

RAW264.7 cells were cultured with a cell-specific medium (DMEM-H + 10%FBS + 1%Glutamax + 1% Sodium Pyruvate + 1%P/S), placed in a 37 °C, 5% CO_2_ incubator. Cells were considered passaged when the cell density was above 90%, and the ratio of cell passages was 1:3.

### 2.8. MTT Assay

RAW264.7 cells were cultured in 96-well plates, and after the cells were attached to the wall, the experimental group was treated with DWS at 20, 60, and 100 µM; the control group was replaced with a fresh medium, and the blank control group had a medium only. After 24 h, 20 μL of MTT (5 mg/mL) solution was added to each well; after 4 h, the solution of each well was discarded, and then 100 μL of DMSO was added, and the reaction was shaken under dark conditions for 10 min. The absorbance values of the groups were determined at 574 nm by a Multi-Mode Microplate Reader. The cell viability of each group was calculated as follows:

$$\mathrm{Cell}\;\mathrm{viability}\;(100\%)=\frac{A_{2}-A_{0}}{A_{1}-A_{0}}\times 100\%$$

where A_0_ represents the absorbance of the blank control group, A_1_ represents the absorbance of the control group, and A_2_ represents the absorbance of the sample group

### 2.9. NO Production Assay

RAW264.7 cells were cultured in 24-well plates. After the cells were attached to the wall, the control and LPS groups were replaced with a new medium, and the experimental group was treated with the peptide DWS at 20, 60, and 100 µM. After culturing for 12 h, the LPS group and the experimental group were treated for 10 h with LPS (1 μg/mL). NO was detected using the Nitric Oxide Assay Kit Reactive Oxygen Detection Kit (Beyotime Biotechnology, Shanghai, China).

### 2.10. Detection of SOD and CAT Activities and MDA Level

RAW264.7 cells were cultured in 6-well cell plates. Cells were grouped and treated as in the NO assay. SOD and CAT activities and the MDA level were detected using the Total Superoxide Dismutase Assay Kit, Catalase (CAT) assay kit, and Malondialdehyde (MDA) assay kit, respectively. The results are expressed as a percentage of the control.

### 2.11. RNA Extraction and qRT-PCR Analysis

RAW264.7 cells were cultured in 6-well cell plates. Cells were grouped and treated as in the NO assay. Cellular RNA extraction was performed with trizol reagent. The RNA was reversely transcribed by using Hifair^®^ AdvanceFast 1st Strand cDNA Synthesis SuperMix for qPCR. Quantitative real-time RT-PCR analysis of related genes was performed by using the Hieff UNICON^®^ Universal Blue qPCR SYBR Green Master Mix. The operating parameters were programmed in accordance with the manufacturer’s instructions. β-actin was used for normalization. The relative gene expression levels were analyzed using the 2^−ΔΔCt^ method. The primer sequences are shown in Table 1.

### 2.12. Western Blot Assay

RAW264.7 cells were cultured in 6-well cell plates. Cells were grouped and treated as in the NO assay. We discarded the cell culture medium from each well and washed each well three times with PBS. We added 1.5 mL of Western and IP cell lysis buffer to harvest the cells and then performed ultrasonication to ensure complete cell disruption. We centrifuged the lysate at 12,000 rpm for 10 min and retained the supernatant. We determined the protein concentration using the BCA assay and adjusted the protein concentrations across samples to uniformity with PBS. We mixed the samples with 5× protein loading buffer at a 1:5 ratio and then denatured them at 100 °C for 10 min using a metal bath. SDS-PAGE gel was used to separate all the proteins and then transferred to a polyvinylidene fluoride (PVDF) membrane. After blocking with 5% skim milk at room temperature for 1 h, the membranes were incubated with primary antibodies (1:1000 dilution) at 4 °C overnight. Following three washes with TBST, secondary antibodies (1:1000 dilution) were applied. In this experiment, β-actin was used as the internal reference protein.

### 2.13. Statistical Analysis

All experiments were performed in at least three independent replicates, with results expressed as mean ± standard deviation. Data were analyzed using one-way analysis of variance (ANOVA) and visualized with GraphPad Prism 8 software. A *p* value < 0.05 was considered statistically significant.

## 3. Results

### 3.1. Virtual Screening of Ulva Prolifera Proteins

A total of 40 *Ulva prolifera* protein amino acid sequences were selected from the NCBI database. *Ulva prolifera* protein sequences were digested in silico by the PeptideCutter program. Peptides containing 2-6 amino acid residues were selected, resulting in the identification of 323 peptides. The potential biological activity and water solubility of these 323 peptides were predicted using the online PeptideRanker and Proteomics tools, respectively. Peptides with activity scores >0.5 and good water solubility were selected. The above peptide sequences were compared with those already included in the BIOPEP-UWM database to remove the reported sequences. Eventually, 120 peptides were obtained (Table 2).

ADMET refers to Absorption, Distribution, Metabolism, Excretion, and Toxicity, which are fundamental parameters for evaluating drug-like properties in pharmaceutical research [41]. The ADMET properties of the above 120 peptides were predicted using admetSAR. Small-molecule drugs are typically administered orally and subsequently absorbed through the intestinal tract. The absorption capacity is commonly evaluated using Caco-2 cell permeability and HIA [42]. The BBB is an endothelial cell barrier that separates the bloodstream from brain tissue. This physiological barrier restricts the passage of most compounds, making BBB permeability a key selection criterion. Additionally, an ideal drug should neither act as a substrate nor an inhibitor of major metabolic enzymes and must exhibit minimal toxicity. Based on the five criteria described above, we screened peptides from the admetSAR-predicted results and identified 19 candidates meeting the requirements (Table 3).

### 3.2. Molecular Docking Analysis

**Figure 1:**
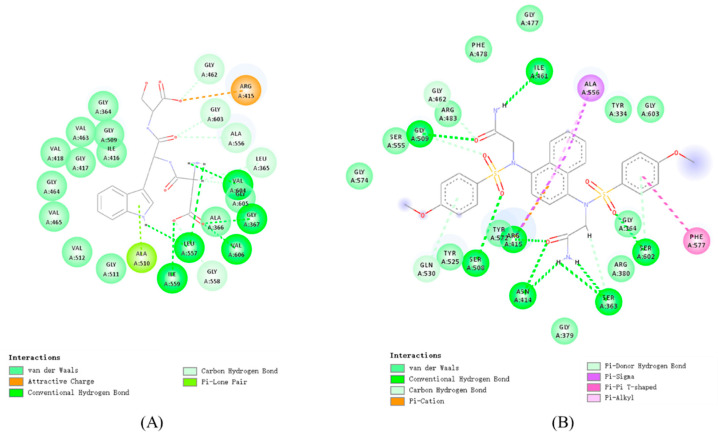
Two-dimensional molecular docking models of (**A**) Keap1-DWS and (**B**) Keap1–2,2′-(naphthalene-1,4-diylbis(((4-methoxyphenyl)sulfonyl)azanediyl))diacetamide.

We performed molecular docking using the “CDOCKER” software in Discovery Studio to determine whether the Keap1 protein binds to the above 19 peptides and the possible binding sites. The binding affinities between the peptides and Keap1 were evaluated using the built-in scoring function “-CDOCKER-ENERGY”. The magnitude of the “-CDOCKER-ENERGY” value is positively correlated with the binding affinity of the peptide–Keap1 complexes [43]. As shown in Table 4, 3 of the 19 peptides obtained from the screening, namely PWFR (Pro-Trp-Phe-Arg), PWYR (Pro-Trp-Tyr-Arg), and YQWD (Tyr-Gln-Trp-Asp), failed to dock with the Keap1 protein, suggesting that these 3 peptides may not interact with the Keap1 protein. Compared to successfully docked di- and tripeptides, these three tetrapeptides failed to dock, probably due to steric hindrance that prevented their accommodation in the Keap1 active region. Among the 16 successfully docked peptides, DWS (Asp-Trp-Ser) had the highest “CDOCKER-ENERGY” values with Keap1, indicating superior binding affinity relative to other candidates. We therefore selected DWS for subsequent studies. Figure 1 presents the 2D docking models of DWS and 2,2′-(naphthalene-1,4-diylbis(((4-methoxyphenyl)sulfonyl)azanediyl))diacetamide with Keap1. The docking results demonstrate that both DWS and 2,2′-(naphthalene-1,4-diylbis(((4-methoxyphenyl)sulfonyl)azanediyl))diacetamide interact with Keap1 through multiple forces: van der Waals interactions, conventional hydrogen bonds, carbon–hydrogen bonds, electrostatic forces, and Pi bonds.

### 3.3. Molecular Dynamics Simulation Analysis

Using the optimal docking conformations of the DWS bound to Keap1, we performed 50 ns MD simulations to characterize the dynamic behavior of the resulting complexes. Structural stability was assessed by analyzing the root mean square deviation (RMSD), root mean square fluctuation (RMSF), solvent-accessible surface area (SASA), and radius of gyration (Rg).

**Figure 2:**
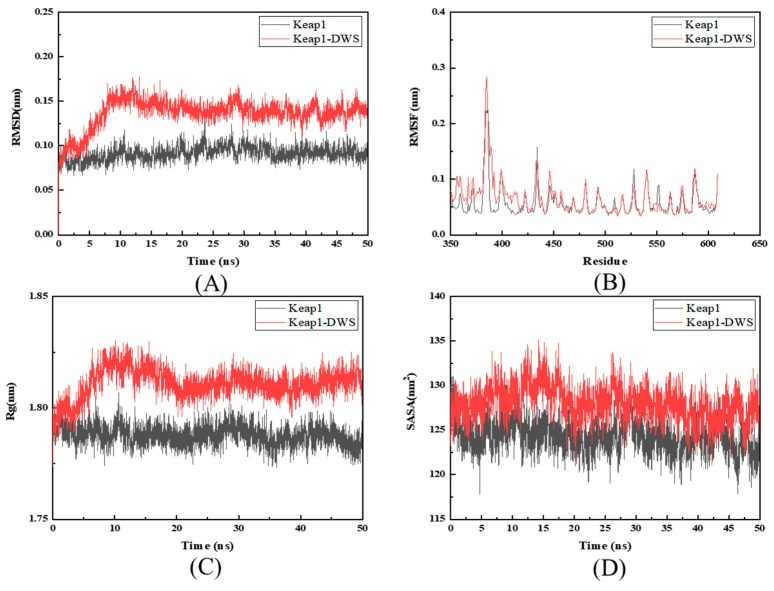
(**A**) RMSD, (**B**) RMSF, (**C**) RG, and (**D**) SASA curves of Keap1 in complex with DWS, as well as of the unloaded Keap1 protein.

The RMSD is the root mean square deviation value of the change in Cα of the backbone of the complex, which can be used to measure the relative change between the conformation of the complex and its initial conformation at any time during the simulation. Lower RMSD values indicate tighter binding interactions and greater conformational stability of the protein–ligand complex. Figure 2A presents the RMSD profiles of DWS-Keap1 complexes and Keap1 over the 50 ns MD simulation. During the initial 10 ns phase, the complexes exhibited progressive increases in RMSD values, Following the initial 10 ns phase, DWS-Keap1 complexes attained a stable RMSD, with Keap1-DWS converging at approximately 0.13 nm, indicating good compatibility between the peptides and the Keap1 protein, resulting in stable complexes. The mean RMSD values over the 50 ns simulation were 0.0913 nm for Keap1 and 0.1366 nm for Keap1-DWS.

The RMSF serves as a critical metric for evaluating conformational dynamics in ligand-protein interaction systems. Lower RMSF values indicate reduced structural perturbation of the receptor protein induced by the ligand, corresponding to enhanced stability of the resulting protein–ligand complex. As shown in Figure 2B, the RMSF profiles of the DWS-Keap1 complexes exhibit highly consistent fluctuation patterns with those of the Keap1 protein. Significant RMSF fluctuations were observed in four regions (residues 360–380, 420–430, 480–500, and 580–590), suggesting potential interactions between these amino acid residues and the small peptide ligands, which may induce conformational flexibility changes. The mean RMSF values of Keap1 and Keap1-DWS were 0.0577 nm and 0.0673 nm, respectively.

The Rg provides a quantitative measure of structural compaction in protein–ligand complexes. Lower Rg values indicate greater structural compaction and consequently higher stability of the protein–ligand complex. Figure 2C presents the radius of gyration (Rg) trajectories of both DWS-Keap1 complexes and Keap1 over the 50 ns simulation period. Consistent with the RMSD trends, the DWS-Keap1 complexes exhibited an initial increase in Rg values during the first 10 ns of simulation, followed by stabilization in the subsequent trajectory. The mean Rg values were determined to be 1.7876 nm for Keap1 and 1.8108 nm for Keap1-DWS.

The SASA represents the total surface area of a protein exposed to the surrounding solvent molecules. Reduced SASA values indicate a more compact and densely packed protein structure, which facilitates ligand binding by creating a hydrophobic environment. Conversely, elevated SASA values reflect a more open conformation that generates a hydrophilic milieu less favorable for ligand interaction. Consequently, a lower SASA correlates with tighter protein–ligand binding and enhanced complex stability. Figure 2D reveals that Keap1 and the DWS-Keap1 complexes maintain highly consistent SASA profiles throughout the 50 ns simulation, with all systems exhibiting fluctuations within the 125–130 nm^2^ range. The mean SASA values of Keap1 and Keap1-DWS were 124.3396 nm^2^ and 128.0148 nm^2^.

### 3.4. DPPH and ABTS Radical Scavenging and Reducing Capacity Analysis

**Figure 3:**
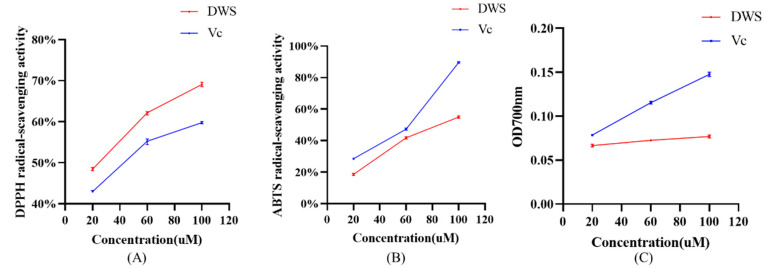
(**A**) DPPH radical scavenging capacity, (**B**) ABTS radical scavenging capacity, and (**C**) reducing capacity of different concentrations of DWS and Vc.

As shown in Figure 3A–C, within the concentration range of 20–100 μM, DWS exhibits dose-dependent scavenging activity against DPPH radicals and ABTS^+^· radicals, as well as reducing capacity. Notably, the DPPH radical scavenging capacity of DWS exceeds that of vitamin C.

### 3.5. RAW264.7 Cell Viability

**Figure 4:**
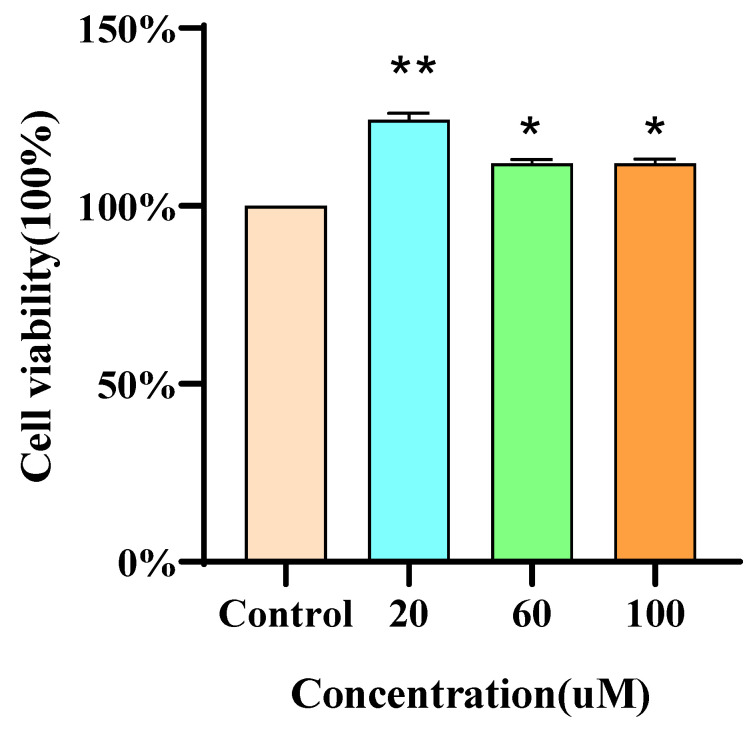
Effect of different concentrations of DWS on the viability of RAW264.7 cells (compared with the control group, * *p* < 0.05, ** *p* < 0.01).

MTT assays revealed that the proliferative effect of DWS on RAW264.7 cells was inversely proportional to its concentration. Importantly, DWS at 20, 60, and 100 μM exhibited no cytotoxicity toward RAW264.7 cells (Figure 4).

### 3.6. Measurement of NO Levels

**Figure 5:**
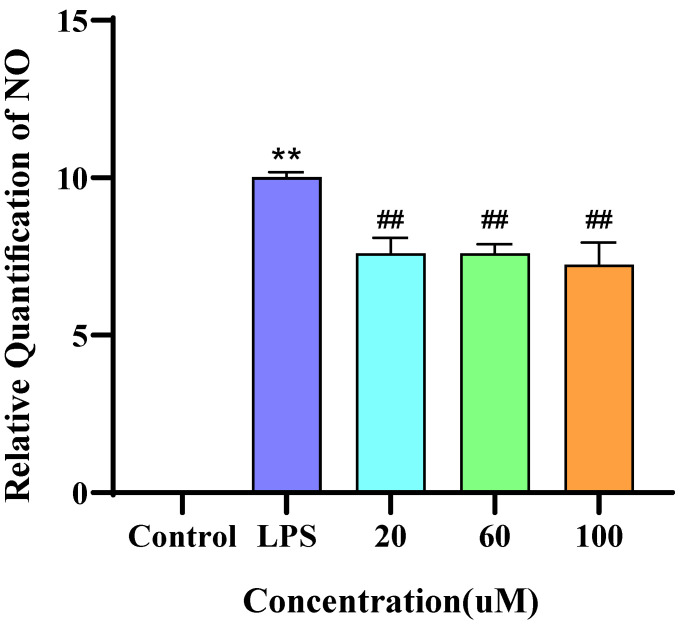
Effect of DWS on NO secretion in RAW264.7 cells (** *p* < 0.01 compared to control group; ## *p* < 0.01 compared to LPS group).

NO levels in the culture supernatant of RAW264.7 cells were measured using the Griess assay. As shown in Figure 5, compared with the control group, LPS treatment for 10 h significantly increased NO production. In contrast, cells treated with low, medium, and high concentrations of DWS exhibited a marked reduction in LPS-induced NO release, with the highest concentration of DWS showing the most potent inhibitory effect.

### 3.7. Effect of DWS on Antioxidant Enzyme Activities and MDA Content in LPS-Induced RAW264.7 Cells

**Figure 6:**
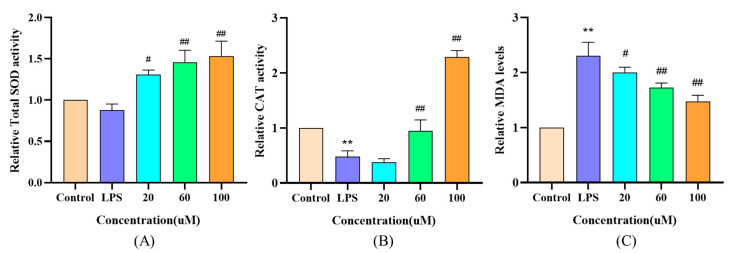
Effect of DWS on (**A**) relative SOD activity, (**B**) relative CAT activity, and (**C**) relative MDA content of RAW264.7 cells (** *p* < 0.01 compared to control group; # *p* < 0.05, ## *p* < 0.01 compared to LPS group).

After LPS treatment, cellular SOD and CAT activities were reduced. As shown in Figure 6A–C, all three concentrations of DWS tested increased SOD activity, whereas only medium and high concentrations increased CAT activity, with both effects showing a dose dependence. LPS treatment significantly increased MDA levels, which were subsequently reduced by DWS treatment in a dose-dependent manner at low, medium, and high concentrations.

### 3.8. Effects of DWS on mRNA Expression of Antioxidant and Anti-Inflammation-Related Genes in RAW264.7 Cells

**Figure 7:**
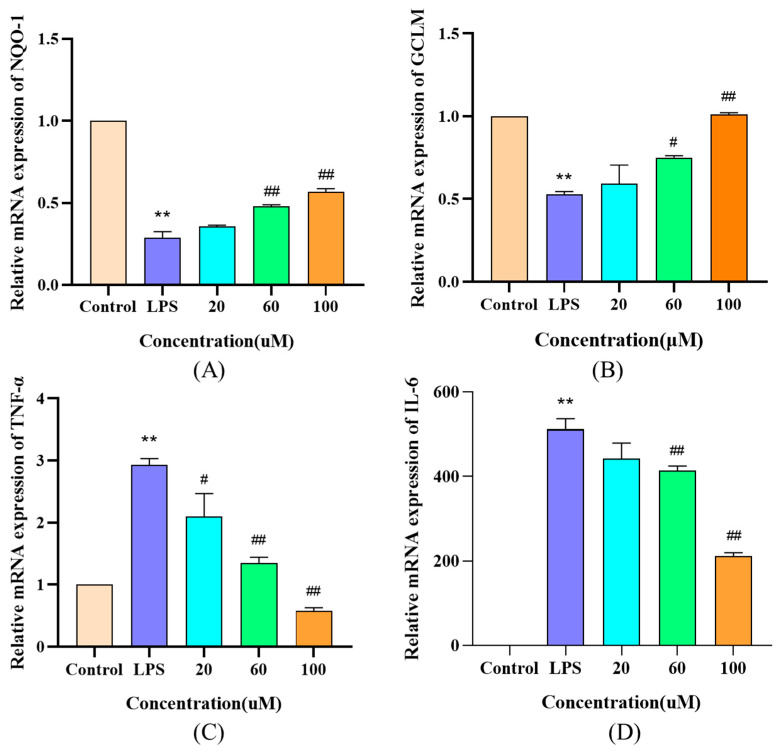
Effects of DWS on mRNA expression of antioxidant and inflammation-related genes in RAW264.7 cells. (**A**) mRNA levels of NQO-1, (**B**) mRNA levels of GCLM, (**C**) mRNA levels of TNF-α, (**D**) mRNA levels of IL-6 (** *p* < 0.01 compared to control group; # *p* < 0.05, ## *p* < 0.01 compared to LPS group).

The mRNA expression levels of antioxidant and inflammation-related genes in RAW264.7 cells were analyzed by q-PCR. As shown in Figure 7, LPS treatment significantly downregulated NQO-1 and GCLM expression compared with the control group. Medium and high concentrations of DWS restored the mRNA expression of these genes, whereas low-concentration DWS showed no significant effect. Following LPS stimulation, the mRNA expression levels of the proinflammatory cytokines TNF-α and IL-6 were significantly elevated compared to the control group. Treatment with varying concentrations of DWS dose-dependently reduced TNF-α and IL-6 mRNA expression.

### 3.9. Western Blot Analysis

**Figure 8:**
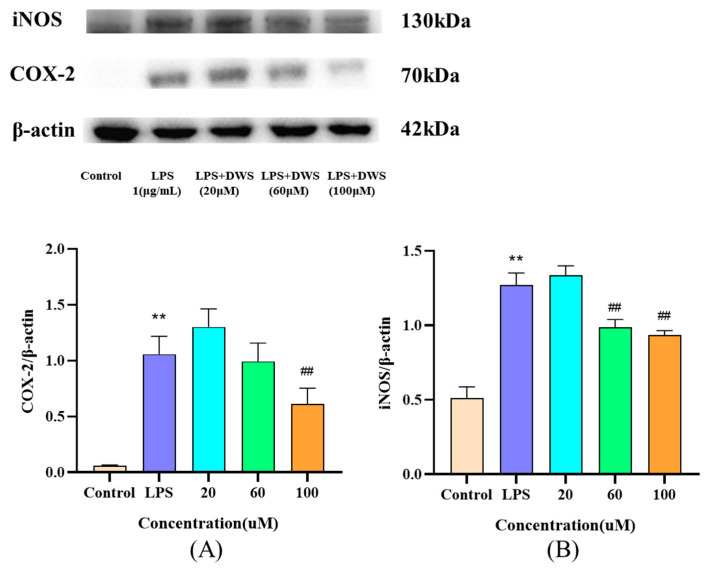
Effects of DWS on the expression of inflammation-related proteins in RAW264.7 cells. (**A**) Protein expression of iNOS, (**B**) protein expression of COX-2 (** *p* < 0.01 compared to control group; ## *p* < 0.01 compared to LPS group).

The expression of the inflammatory proteins iNOS and COX-2 in RAW264.7 cells was analyzed by Western blotting. As shown in Figure 8, LPS stimulation significantly upregulated both iNOS and COX-2 protein levels compared to the control group. Notably, while medium and high concentrations of DWS significantly attenuated LPS-induced iNOS expression, only high-dose DWS effectively reduced COX-2 protein levels.

## 4. Discussion

OS is a state in which the oxidative capacity of an organism exceeds its antioxidant capacity, resulting from an increase in reactive oxygen species production, a decrease in antioxidant capability, or a combination of both [44]. OS can also regulate inflammation by activating the expression of cytokines and chemokines induced by the transcription factor nuclear factor κB (NF-κB) [45]. The supplementation of exogenous antioxidants has been demonstrated to be an effective countermeasure against oxidative stress. Bioactive peptides, derived from food proteins, have been shown to be efficacious exogenous antioxidants. Numerous studies have been conducted to prepare bioactive peptides with antioxidant and anti-inflammatory effects from food proteins [11,12]. *Ulva prolifera*, an edible green alga, exhibits high protein content and a diverse amino acid profile. Studies have shown that Ulva proteins can be hydrolyzed to generate peptide derivatives with biological activity [32]. Computer-aided virtual screening has been widely employed in the preparation of various bioactive peptides due to its cost-effectiveness and high efficiency [46]. In this study, we employed in silico proteolysis using pepsin and trypsin to simulate gastrointestinal digestion of *Ulva prolifera*-derived protein sequences, generating numerous peptide fragments. Through bioactivity screening and ADMET profiling, we identified 19 candidate peptides. Both bioactivity and good water solubility are critical for small peptides to exert physiological effects [47] (Dinesh et al., 2024), while ADMET properties are essential parameters for pharmacological evaluation [41].

Keap1 is a negative regulator of Nrf2. Disruption of the Keap1-Nrf2 interaction facilitates Nrf2 release and subsequent nuclear translocation, thereby activating downstream antioxidant response elements [48]. Therefore, investigating the interaction between antioxidant compounds and Keap1 represents an effective strategy for exploring their potential to enhance the endogenous antioxidant system. Molecular docking is an effective tool for studying the interactions between proteins and their ligands. After identifying 19 candidate peptides, we performed molecular docking with the Keap1 protein to characterize its potential binding interactions. The docking results revealed that only 16 peptides successfully bound to Keap1, with the peptide DWS exhibiting the highest ‘CDOCKER_ENERGY’ value, indicating its strongest binding affinity. DWS interacts with Keap1 primarily through van der Waals forces, hydrogen bonds, electrostatic interactions, and π-stacking, with hydrogen bonding identified as the dominant interaction, which is consistent with the results of Zhuang et al. [49]. 2,2′-(naphthalene-1,4-diylbis(((4-methoxyphenyl)sulfonyl)azanediyl))diacetamide acts as a potent Nrf2 activator [50]. Molecular docking results indicate that both DWS and 2,2′-(naphthalene-1,4-diylbis(((4-methoxyphenyl)sulfonyl)azanediyl))diacetamide can bind with ACE at several sites: Gly364, Gly462, Gly603, Ala556, Arg415. Studies by Xu et al., Li et al., and Yao [21,22,23] have demonstrated that certain antioxidant compounds bind to Keap1 via residues Leu557, Gly558, Ile559, Val604, and Val606. In our results, DWS was found to form hydrogen bonds with these specific Keap1 amino acid residues. Additionally, DWS forms an electrostatic interaction with Arg415 of Keap1. Notably, Arg415 of Keap1 is considered one of the key residues involved in its binding to Nrf2 [51]. Collectively, these conserved amino acid residues may delineate key interaction sites between DWS and Keap1, suggesting the potential antioxidant activity of DWS. Further molecular dynamics simulations were employed to investigate the dynamic binding process of DWS with Keap1. The simulation results were evaluated using RMSD, RMSF, SASA, and Rg values. An analysis revealed that, compared to Keap1 alone, the DWS-Keap1 complex exhibited moderate fluctuations but remained stable overall during the 50 ns simulation. This stability likely arises from extensive van der Waals forces and hydrogen bonds formed between DWS and multiple amino acid residues within Keap1’s binding pocket [52,53], which help maintain the complex’s structural integrity. These findings further support the interaction between DWS and Keap1, suggesting its potential antioxidant capacity.

Chemical-based assays are commonly used for in vitro evaluation of antioxidant activity. These methods can be categorized by their underlying chemical reactions into two types: hydrogen atom transfer (HAT)-based assays and electron transfer (ET- based assays [54]. In studies of antioxidant peptides, ET-based assays are routinely employed for the initial assessment of their antioxidant capacity [55,56]. We therefore further measured the DPPH and ABTS radical scavenging capacity and reducing capacity of DWS. The results demonstrated that DWS exhibits strong DPPH and ABTS radical scavenging activity and reducing capacity. According to Chen et al. [57], antioxidant peptides typically contain antioxidative amino acid residues. Notably, DWS contains the antioxidative amino acids Asp and Trp, which may account for its potent in vitro antioxidant activity.

In cellular oxidative stress models, antioxidant enzyme activity and malondialdehyde (MDA) levels are commonly used as biomarkers [58]. Antioxidant enzymes serve as the first line of defense against cellular oxidative stress, including SOD and CAT. SOD scavenges superoxide radicals, while CAT eliminates hydrogen peroxide [59]. MDA is a cellular marker of lipid peroxidation, and higher levels of MDA in cells indicate greater oxidative damage to cells [60]. Heme oxygenase-1 (HO-1) and recombinant glutamate–cysteine ligase (GCLM) are key downstream target genes regulated by Nrf2, playing critical roles in protecting cells against inflammatory responses and oxidative damage [61].

Upon stimulation, immune cells secrete cytokines as intercellular signaling mediators to regulate immune responses and mediate inflammatory reactions [62]. For example, macrophages produce inflammatory cytokines such as NO, TNF-α, and IL-6 when excessively stimulated by LPS [63]. Among these, NO is a crucial inflammatory mediator. Excessive NO production may lead to the generation of reactive nitrogen species, which can react with biological macromolecules and cause cellular damage [64]. TNF-α is the most potent proinflammatory cytokine, capable of promoting leukocyte adhesion to epithelium through adhesion molecule expression and enhancing oxidative stress at inflammatory sites [65,66]. IL-6, abundantly secreted during early inflammation, acts as an inflammatory amplifier that regulates immune cell proliferation and differentiation while enhancing natural killer cell activity [67]. These cytokines are interconnected, collectively forming an inflammatory network [68]. In addition to the aforementioned cytokines, iNOS and COX-2 play crucial roles in inflammatory responses. iNOS serves as the rate-limiting enzyme for NO synthesis, while COX-2 induces PGE2 production. LPS stimulation upregulates the expression of both iNOS and COX-2 proteins in RAW264.7 cells [69,70].

In our study, DWS increased SOD and CAT activities while decreasing MDA levels and NO secretion in RAW264.7 cells. Furthermore, DWS upregulated the mRNA expression of antioxidant-related genes (NQO-1 and GCLM) and downregulated proinflammatory genes (TNF-α and IL-6), while suppressing the expression of inflammatory proteins (iNOS and COX-2). These results demonstrate that DWS protects against LPS-induced oxidative stress in RAW264.7 cells, exhibiting both antioxidant and anti-inflammatory activities, consistent with findings from Lu et al. and Zhao et al. (2016) [71,72]. While our in vitro findings demonstrate DWS’s antioxidant and anti-inflammatory properties, future studies in animal models are needed to verify these protective effects at the organism level. Extensive research has established that protein hydrolysates containing abundant short-chain peptides, particularly di- and tripeptides, exhibit superior intestinal absorption efficiency compared to both free amino acids and longer precursor peptides [73]. The tripeptide’s small size and natural amino acid composition suggest good potential as a functional food ingredient, though its stability during gastrointestinal digestion and oral bioavailability require systematic evaluation.

## 5. Conclusions

In this study, we identified a novel antioxidant and anti-inflammatory peptide derived from *Ulva prolifera* protein using virtual screening techniques. Specifically, the potential antioxidant peptide DWS was screened through in silico enzymatic hydrolysis, virtual screening, molecular docking, and MD simulations. Its functional effects were further validated by in vitro antioxidant assays, MTT assays, enzymatic activity measurements, qPCR, and Western blot analysis. Molecular docking and dynamics simulations revealed that DWS forms stable complexes with Keap1 by establishing hydrogen bonds and Pi bonds with conserved amino acid residues (Leu557, Gly558, Ile559, Val604, Val606, and Arg415). Further studies demonstrated that DWS not only exhibits strong DPPH and ABTS radical scavenging activity and reducing power but also enhances SOD and CAT activity in RAW264.7 cells while reducing MDA levels and NO secretion. Additionally, DWS upregulates GCLM and HO-1 gene expression while downregulating IL-6, TNF-α, and COX-2 expression at both the mRNA and protein levels. These findings suggest that DWS protects RAW264.7 cells against LPS-induced oxidative damage, exhibiting significant antioxidant and anti-inflammatory effects. This study provides a theoretical foundation for the utilization of *Ulva prolifera* resources and the development of antioxidant and anti-inflammatory peptides.

## Figures and Tables

**Table 1:** The primer sequences.

Gene	Forward/Reverse	Primer Sequence
β-actin	Forward	CGGTTCCGATGCCCTGAGGCTCTT
	Reverse	CGTCACACTTCATGATGGAATTGA
NQO-1	Forward	TTCTCTGGCCGATTCAGAGTG
	Reverse	TCAGCTGGAATGGACTTGCC
HO-1	Forward	CCCACCAAGTTCAAACAGCTC
	Reverse	AGGAAGGCGGTCTTAGCCTC
GCLM	Forward	ATGACCCGAAAGAACTGCTC
	Reverse	CTGCTCTTCACGATGACCGA
TNF-α	Forward	ACCCTCACACTCACAAACCA
	Reverse	ATAGCAAATCGGCTGACGGT
IL-6	Forward	GGTGACAACCACGGCCTTCCC
	Reverse	AAGCCTCCGACTTGTGAAGTGGT

**Table 2:** Peptides obtained after virtual enzymatic digestion, bioactivity, and water solubility selection.

Peptide	Activity Score	Water Solubility	Peptide	Activity Score	Water Solubility	Peptide	Activity Score	Water Solubility
FR	0.985719	good	FK	0.860392	good	EF	0.598976	good
WR	0.977891	good	MR	0.849148	good	LR	0.569984	good
WK	0.866241	good	PR	0.787626	good	CQ	0.540359	good
CR	0.865233	good	GR	0.766288	good	YR	0.525242	good
FMR	0.980845	good	DDF	0.748981	good	LGR	0.620526	good
FPR	0.976464	good	DQF	0.736087	good	YDC	0.609016	good
MDF	0.959246	good	NDF	0.723384	good	DPR	0.599321	good
FDP	0.930726	good	FTR	0.706612	good	FNK	0.592861	good
GCR	0.902065	good	AWK	0.698525	good	MGK	0.584511	good
FIR	0.893444	good	CDG	0.68045	good	NFK	0.577538	good
DFL	0.889906	good	YGR	0.676333	good	GPK	0.567438	good
DWR	0.876857	good	SWK	0.666979	good	FEL	0.566207	good
FKP	0.871158	good	MIR	0.66231	good	EFR	0.557213	good
YDF	0.870185	good	GSP	0.653894	good	FSK	0.556465	good
LDF	0.839471	good	QCR	0.643288	good	MSR	0.554004	good
FSR	0.831241	good	CKP	0.642963	good	DVF	0.551408	good
GGR	0.81127	good	DWS	0.637383	good	GDL	0.546664	good
FLK	0.805333	good	MAR	0.636319	good	FER	0.529433	good
YMR	0.780662	good	GCK	0.632386	good	DGL	0.525785	good
FYK	0.764839	good	IPR	0.629111	good	PGK	0.517877	good
YPR	0.754834	good	FAK	0.621611	good	SGR	0.508729	good
PWFR	0.990371	good	QPPR	0.762773	good	MYHR	0.587398	good
PWYR	0.942612	good	FQPK	0.72783	good	FLIK	0.582359	good
FIPR	0.912571	good	YQWD	0.687401	good	FNYK	0.577253	good
IPFR	0.906606	good	GSMR	0.675175	good	FHYK	0.572896	good
PGMR	0.900324	good	DMHP	0.639519	good	TFPK	0.561414	good
CDGC	0.839955	good	DCYR	0.631674	good	KPEF	0.557678	good
FMTR	0.821677	good	CCDA	0.625188	good	GGNR	0.546288	good
KPDF	0.810801	good	FGNK	0.601468	good	LGPK	0.516678	good
HCPR	0.800373	good	FKPK	0.592118	good	FIIK	0.506281	good
FKPDL	0.802916	good	LDPCR	0.727549	good	DDSMC	0.586729	good
DWIGR	0.789988	good	FISGR	0.723567	good	NKPVF	0.583004	good
QFPNR	0.772362	good	SCPQR	0.680029	good	YECGL	0.543409	good
EGCAW	0.761261	good	FAPAK	0.672649	good	NDPGR	0.531025	good
WSPEL	0.736445	good	AMDGL	0.640645	good	GPAAR	0.515691	good
FSPIK	0.730555	good	LDPAL	0.595302	good	YGPTR	0.514815	good
DDYHC	0.504222	good	FDGNMP	0.863932	good	FLPPNK	0.744351	good
SWNTPR	0.739203	good	WAKPGH	0.688682	good	SGMCHD	0.559215	good
FLKPDL	0.711564	good	GRPADM	0.636649	good	SGKPAL	0.540218	good
AWDSYR	0.692275	good	PEPWAK	0.578204	good	DGLNPR	0.50385	good

**Table 3:** Small-molecule peptides obtained after ADMET selection.

Peptides	Caco2-	BBB+	HIA+	Metabolism or Inhibitor	Toxicity
	**Probability**				
MR	0.6739	0.7091	0.8599	non	non
PR	0.7649	0.7597	0.6889	non	non
LR	0.752	0.698	0.8271	non	non
YR	0.8827	0.6121	0.6163	non	non
FR	0.7607	0.7849	0.7912	non	non
WR	0.762	0.8368	0.9644	non	non
WK	0.7574	0.8534	0.9708	non	non
FK	0.7064	0.8011	0.8238	non	non
AWK	0.8394	0.6041	0.9441	non	non
DWS	0.8544	0.7165	0.7926	non	non
DWR	0.8101	0.6244	0.7047	non	non
MAR	0.7039	0.5787	0.5799	non	non
MGK	0.685	0.683	0.6725	non	non
FAK	0.8181	0.6068	0.7042	non	non
FYK	0.8468	0.5196	0.7024	non	non
FEL	0.8278	0.5648	0.5695	non	non
PWFR	0.822	0.5349	0.8572	non	non
PWYR	0.861	0.6014	0.8955	non	non
YQWD	0.8771	0.5461	0.6572	non	non

**Table 4:** Molecular docking results of selected peptides with Keap1 protein.

Peptides	CDOCKER-ENERGY (kcal/mol)	Peptides	CDOCKER-ENERGY (kcal/mol)
DWS	88.4352	LR	71.0558
DWR	87.7048	YR	71.0558
MGK	87.4723	FR	68.3871
FAK	87.175	WR	67.3156
FEL	80.9024	MR	67.1224
FYK	79.266	PR	42.9183
MAR	73.7247	PWFR	No
FK	73.5543	PWYR	No
AWK	72.8721	YQWD	No
WK	71.1864		

No: Indicates that the small peptide did not successfully dock with the Keap1 protein.

## Data Availability

The original contributions presented in this study are included in the article. Further inquiries can be directed to the corresponding author.

## Supplementary Materials

The following are available online at https://www.mdpi.com/article/10.3390/foods14132202/s1, Table S1: *Ulva prolifera* protein selected from the NCBI database.

## References

[1] Li Z., Xu X., Leng X., He M., Wang J., Cheng S., Wu H. (2017). Roles of reactive oxygen species in cell signaling pathways and immune responses to viral infections. Arch. Virol..

[2] Pérez-Torres I., Castrejón-Téllez V., Soto M.E., Rubio-Ruiz M.E., Manzano-Pech L., Guarner-Lans V. (2021). Oxidative Stress, Plant Natural Antioxidants, and Obesity. Int. J. Mol. Sci..

[3] Estévez M. (2011). Protein carbonyls in meat systems: A review. Meat Sci..

[4] Lieber M.R., Karanjawala Z.E. (2004). Ageing, repetitive genomes and DNA damage. Nat. Rev. Mol. Cell Biol..

[5] Forman H.J., Zhang H. (2021). Targeting oxidative stress in disease: Promise and limitations of antioxidant therapy. Nat. Rev. Drug Discov..

[6] Dodson M., de la Vega M.R., Cholanians A.B., Schmidlin C.J., Chapman E., Zhang D.D. (2019). Modulating NRF_2_ in Disease: Timing Is Everything. Annu. Rev. Pharmacol..

[7] Taguchi K., Yamamoto M. (2020). The KEAP1-NRF_2_ System as a Molecular Target of Cancer Treatment. Cancers.

[8] Cloer E.W., Siesser P.F., Cousins E.M., Goldfarb D., Mowrey D.D., Harrison J.S., Weir S.J., Dokholyan N.V., Major M.B. (2018). p62-Dependent Phase Separation of Patient-Derived KEAP_1_ Mutations and NRF_2_. Mol. Cell. Biol..

[9] Saadi S., Saari N., Anwar F., Abdul Hamid A., Ghazali H.M. (2015). Recent advances in food biopeptides: Production, biological functionalities and therapeutic applications. Biotechnol. Adv..

[10] Taniguchi M., Aida R., Saito K., Ochiai A., Takesono S., Saitoh E., Tanaka T. (2018). Identification and characterization of multifunctional cationic peptides from traditional Japanese fermented soybean Natto extracts. J. Biosci. Bioeng..

[11] Du Y., Esfandi R., Willmore W.G., Tsopmo A. (2016). Antioxidant Activity of Oat Proteins Derived Peptides in Stressed Hepatic HepG_2_ Cells. Antioxidants.

[12] Jin D.X., Liu X.L., Zheng X.Q., Wang X.J., He J.F. (2016). Preparation of antioxidative corn protein hydrolysates, purification and evaluation of three novel corn antioxidant peptides. Food Chem..

[13] Samaei S.P., Ghorbani M., Tagliazucchi D., Martini S., Gotti R., Themelis T., Tesini F., Gianotti A., Gallina Toschi T., Babini E. (2020). Functional, nutritional, antioxidant, sensory properties and comparative peptidomic profile of faba bean (*Vicia faba*, L.) seed protein hydrolysates and fortified apple juice. Food Chem..

[14] Luo X., Fei Y., Xu Q., Lei T., Mo X., Wang Z., Zhang L., Mou X., Li H. (2020). Isolation and identification of antioxidant peptides from tartary buckwheat albumin (*Fagopyrum tataricum* Gaertn.) and their antioxidant activities. J. Food Sci..

[15] Liu X., Luo T.J., Han L.J., Gui L.S., Hou S.Z., Sun S.G., Zhao C.Z. (2024). Simulated enzymatic screening and activity validation of antioxidant peptides from Tibetan sheep hemoglobin. Food Ferment. Ind..

[16] Chen Y.N., Qiu Z.J., Zhang B. (2024). Virtual Screening and Molecular Docking of Antioxidant Peptides Derived from Walnut Proteins. Agric. Prod. Process..

[17] Li J.C., Gao X.Y., Jiang C.Y., Yang Y.S., Saimaiti A., Liu Y.N., Batuer A. (2023). Simulation of Enzymolysis to Optimize the Preparation of Pigeon Hemoglobin Antioxidant Peptides. Food Res. Dev..

[18] Wani T.A., Zargar S., Hussain A. (2022). Spectroscopic, Thermodynamic and Molecular Docking Studies on Molecular Mechanisms of Drug Binding to Proteins. Molecules.

[19] Tuckerman M.E., Martyna G.J. (2001). Understanding modern molecular dynamics: Techniques and applications. J. Phys. Chem. B.

[20] Khan A., Ali S.S., Khan M.T., Saleem S., Ali A., Suleman M., Babar Z., Shafiq A., Khan M., Wei D.Q. (2021). Combined drug repurposing and virtual screening strategies with molecular dynamics simulation identified potent inhibitors for SARS-CoV-2 main protease (3CLpro). J. Biomol. Struct. Dyn..

[21] Xu Y., Song D., Su Y., Chen J., Wu L., Lian H., Hai N., Li J., Jiang J., Zhao J. (2023). Pharmacology-based molecular docking of 4-methylcatechol and its role in RANKL-mediated ROS/Keap_1_/Nrf_2_ signalling axis and osteoclastogenesis. Biomed. Pharmacother..

[22] Li M., Huang W., Jie F., Wang M., Zhong Y., Chen Q., Lu B. (2019). Discovery of Keap_1_-Nrf_2_ small-molecule inhibitors from phytochemicals based on molecular docking. Food Chem. Toxicol..

[23] Yao H., He Q., Huang C., Wei S., Gong Y., Li X., Liu W., Xu Z., Wu H., Zheng C. (2022). Panaxatriol saponin ameliorates myocardial infarction-induced cardiac fibrosis by targeting Keap_1_/Nrf_2_ to regulate oxidative stress and inhibit cardiac-fibroblast activation and proliferation. Free Radic. Biol. Med..

[24] Tonolo F., Moretto L., Grinzato A., Fiorese F., Folda A., Scalcon V., Ferro S., Arrigoni G., Bellamio M., Feller E. (2020). Fermented Soy-Derived Bioactive Peptides Selected by a Molecular Docking Approach Show Antioxidant Properties Involving the Keap_1_/Nrf_2_ Pathway. Antioxidants.

[25] Ding L., Luan R., Huang B. (2008). The taxonomical study on Capsosiphonaceae (Ulvales, Chlorophyta) from Huanghai-Bohai Seas of China. Acta Oceanol. Sin..

[26] Jiang X.L., Zhou X.J., Lin J.N., Kang Z.J., Liu Q. (2021). Research progress in the ecological consequences of *Ulva prolifera* green tides in the Yellow Sea. Mar. Environ. Sci..

[27] Gao S., Chen X., Yi Q., Wang G., Pan G., Lin A., Peng G. (2010). Astrategy for the proliferation of *Ulva prolifera*, main causative species of tides, with formation of sporangia by fragmentation. PLoS ONE.

[28] Li J.Y., Yang F., Jin L., Wang Q., Yin J., He P., Chen Y. (2018). Safety and quality of the green tide algal species *Ulva prolifera* for option of human consumption: A nutrition and contamination study. Chemosphere.

[29] Dhargalkar V.K., Pereira N. (2005). Seaweed: Promising plant of the millennium. Sci. Cul..

[30] Echave J., Fraga-Corral M., Garcia-Perez P., Popović-Djordjević J., Avdović E.H., Radulović M., Xiao J., Prieto M.A., Simal-Gandara J. (2021). Seaweed Protein Hydrolysates and Bioactive Peptides: Extraction, Purification, and Applications. Mar. Drugs.

[31] Pimentel F.B., Cermeño M., Kleekayai T., Harnedy-Rothwell P.A., Fernandes E., Alves R.C., Oliveira M.B.P.P., FitzGerald R.J. (2020). Enzymatic Modification of Porphyra dioica-Derived Proteins to Improve their Antioxidant Potential. Molecules.

[32] Li Z., He Y., He H., Zhou W., Li M., Lu A., Che T., Shen S. (2023). Purification identification and function analysis of ACE inhibitory peptide from *Ulva prolifera* protein. Food Chem..

[33] Wiederschain G.Y. (2006). The proteomics protocols handbook. Biochemistry (Moscow).

[34] Mooney C., Haslam N.J., Pollastri G., Shields D.C. (2012). Towards the improved discovery and design of functional peptides: Common features of diverse classes permit generalized prediction of bioactivity. PLoS ONE.

[35] Minkiewicz P., Iwaniak A., Darewicz M. (2019). BIOPEP-UWM Database of Bioactive Peptides: Current Opportunities. Int. J. Mol. Sci..

[36] Cheng F., Li W., Zhou Y., Shen J., Wu Z., Liu G., Lee P.W., Tang Y. (2012). AdmetSAR: A comprehensive source and free tool for assessment of chemical ADMET properties. J. Chem. Inf. Model..

[37] Burley S.K., Berman H.M., Kleywegt G.J., Markley J.L., Nakamura H., Velankar S. (2017). Protein Data Bank (PDB): The Single Global Macromolecular Structure Archive. Methods Mol. Biol..

[38] Liu J., Wang C., Wang Z., Zhang C., Lu S., Liu J. (2011). The antioxidant and free-radical scavenging activities of extract and fractions from corn silk (*Zea mays* L.) and related flavone glycosides. Food Chem..

[39] Liu R., Zheng W., Li J., Wang L., Wu H., Wang X., Shi L. (2015). Rapid identification of bioactive peptides with antioxidant activity from the enzymatic hydrolysate of *Mactra veneriformis* by UHPLC-Q-TOF mass spectrometry. Food Chem..

[40] Thaipong K., Boonprakob U., Crosby K., Cisneros-Zevallos L., Byrne D.H. (2012). Comparison of ABTS, DPPH, FRAP, and ORAC assays for estimating antioxidant activity from guava fruit extracts. J. Food Compos. Anal..

[41] Ferreira L.L.G., Andricopulo A.D. (2019). ADMET modeling approaches in drug discovery. Drug Discov. Today.

[42] Hou T., Wang J., Li Y. (2007). ADME evaluation in drug discovery.8. The prediction of human intestinal absorption by a support vector machine. J. Chem. Inf. Model..

[43] Yu Z., Kan R., Wu S., Guo H., Zhao W., Ding L., Zheng F., Liu J. (2021). Xanthine oxidase inhibitory peptides derived from tuna protein: Virtual screening, inhibitory activity, and molecular mechanisms. J. Sci. Food Agric..

[44] Duni A., Liakopoulos V., Roumeliotis S., Peschos D., Dounousi E. (2019). Oxidative Stress in the Pathogenesis and Evolution of Chronic Kidney Disease: Untangling Ariadne’s Thread. Int. J. Mol. Sci..

[45] Stenvinkel P., Chertow G.M., Devarajan P., Levin A., Andreoli S.P., Bangalore S., Warady B.A. (2021). Chronic Inflammation in Chronic Kidney Disease Progression: Role of Nrf_2_. Kidney Int. Rep..

[46] Chen J., Yu X., Chen Q., Wu Q., He Q. (2022). Screening and mechanisms of novel angiotensin-I-converting enzyme inhibitory peptides from rabbit meat proteins: A combined in silico and in vitro study. Food Chem..

[47] Dinesh S., Sharma S., Chourasiya R. (2024). Therapeutic Applications of Plant and Nutraceutical-Based Compounds for the Management of Type 2 Diabetes Mellitus: A Narrative Review. Curr. Diabetes Rev..

[48] Suzuki T., Yamamoto M. (2015). Molecular basis of the Keap1-Nrf2 system. Free Radic. Biol. Med..

[49] Zhuang C., Narayanapillai S., Zhang W., Sham Y.Y., Xing C. (2014). Rapid identification of Keap1-Nrf2 small-molecule inhibitors through structure-based virtual screening and hit-based substructure search. J. Med. Chem..

[50] Jain A.D., Potteti H., Richardson B.G., Kingsley L., Luciano J.P., Ryuzoji A.F., Lee H., Krunic A., Mesecar A.D., Reddy S.P. (2015). Probing the structural requirements of non-electrophilic naphthalene-based Nrf2 activators. Eur. J. Med. Chem..

[51] Lo S.C., Li X., Henzl M.T., Beamer L.J., Hannink M. (2006). Structure of the Keap1:Nrf2 interface provides mechanistic insight into Nrf2 signaling. EMBO J..

[52] Liu W., Liu R., Qin Q., Wang H., Zhang X., Meng G. (2024). Molecular docking and molecular dynamics simulation of wheat gluten-derived antioxidant peptides acting through the Keap1-Nrf2 pathway. J. Sci. Food. Agric..

[53] Wilson C.J., Chang M., Karttunen M., Choy W.Y. (2021). KEAP_1_ Cancer Mutants: A Large-Scale Molecular Dynamics Study of Protein Stability. Int. J. Mol. Sci..

[54] Huang D., Ou B., Prior R.L. (2005). The chemistry behind antioxidant capacity assays. J. Agric. Food Chem..

[55] Yang X.R., Zhao Y.Q., Qiu Y.T., Chi C.F., Wang B. (2019). Preparation and characterization of gelatin and antioxidant peptides from gelatin hydrolysate of Skipjack Tuna (*Katsuwonus pelamis*) bone stimulated by in vitro gastrointestinal digestion. Mar. Drugs.

[56] Farzaneh R.S.A. (2018). Bioactive properties of *Agaricus bisporus* and *Terfezia claveryi* proteins hydrolyzed by gastrointestinal proteases. Lebensm. Wiss. Technol..

[57] Chen J., Yan Y., Zhang L., Zheng J., Guo J., Li R., Zeng J. (2021). Purification of novel antioxidant peptides from myofibrillar protein hydrolysate of chicken breast and their antioxidant potential in chemical and H_2_O_2_-stressed cell systems. Food Funct..

[58] Aguilar-Toalá J.E., Liceaga A.M. (2021). Cellular antioxidant effect of bioactive peptides and molecular mechanisms underlying: Beyond chemical properties. Int. J. Food Sci. Technol..

[59] Ighodaro O.M., Akinloye O.A. (2018). First line defence antioxidants-superoxide dismutase (SOD), catalase (CAT) and glutathione peroxidase (GPX): Their fundamental role in the entire antioxidant defence grid. Alex J. Med..

[60] Yu Q., Tao Y., Huang Y., Zogona D., Wu T., Liu R., Pan S., Xu X. (2022). Aged Pericarpium Citri Reticulatae ‘Chachi’ Attenuates Oxidative Damage Induced by tert-Butyl Hydroperoxide (t-BHP) in HepG2 Cells. Foods.

[61] Wu Y., Zhao Y., Yang H.Z., Wang Y.J., Chen Y. (2021). HMGB1 regulates ferroptosis through Nrf2 pathway in mesangial cells in response to high glucose. Biosci. Rep..

[62] Zhou M., Tang Y., Liao L., Liu M., Deng Y., Zhao X., Li Y. (2021). Phillygenin inhibited LPS-induced RAW 264.7 cell inflammation by NF-κB pathway. Eur. J. Pharmacol..

[63] Huang D., Chen Y., Chen W., Liu Y., Yao F., Xue D., Sun L. (2015). Anti-inflammatory effects of the extract of Gnaphalium affine D Don in vivo and in vitro. J. Ethnopharmacol..

[64] Boscá L., Zeini M., Través P.G., Hortelano S. (2005). Nitric oxide and cell viability in inflammatory cells: A role for NO in macrophage function and fate. Toxicology.

[65] Beutler B., Cerami A. (1987). Cachectin: More than a tumor necrosis factor. N. Engl. J. Med..

[66] Moreira-Tabaka H., Peluso J., Vonesch J.L., Hentsch D., Kessler P., Reimund J.M., Dumont S., Muller C.D. (2012). Unlike for human monocytes after LPS activation, release of TNF-α by THP-1 cells is produced by a TACE catalytically different from constitutive TACE. PLoS ONE.

[67] Hirano T. (2010). Interleukin 6 in autoimmune and inflammatory diseases: A personal memoir. Proceedings of the Japan Academy. Proc. Jpn. Acad. Ser. B Phys. Biol. Sci..

[68] Pan W., Wang Q., Chen Q. (2019). The cytokine network involved in the host immune response to periodontitis. Int. J. Oral Sci..

[69] Yuan Q., Wang J., Guo L., Xu Y., Hu L., Mao H., Miao L., Zhang H., Chai L. (2022). Neobavaisoflavone ameliorates LPS-induced RAW264.7 cell inflammations by suppressing the activation of NF-κB and MAPKs signaling pathways. Iran J. Basic Med. Sci..

[70] Zhang X., Xiong H., Li H., Yu L., Deng X. (2011). Effects of florfenicol on LPS-induced nitric oxide and prostaglandin E_2_ production in RAW 264.7 macrophages. Fundam. Clin. Pharmacol..

[71] Lu M.C., Jiao Q., Liu T., Tan S.J., Zhou H.S., You Q.D., Jiang Z.Y. (2018). Discovery of a head-to-tail cyclic peptide as the Keap_1_-Nrf_2_ protein-protein interaction inhibitor with high cell potency. Eur. J. Med. Chem..

[72] Zhao L., Wang X., Zhang X.L., Xie Q.F. (2016). Purification and identification of anti-inflammatory peptides derived from simulated gastrointestinal digests of velvet antler protein (*Cervus elaphus* Linnaeus). J. Food Drug Anal..

[73] Abeer M.M., Trajkovic S., Brayden D.J. (2021). Measuring the oral bioavailability of protein hydrolysates derived from food sources: A critical review of current bioassays. Biomed. Pharmacother..

