# Supplementary material for: Antioxidant Activity In Vitro and Protective Effects Against Lipopolysaccharide-Induced Oxidative Stress and Inflammation in RAW264.7 Cells of Ulva prolifera-Derived Bioactive Peptides Identified by Virtual Screening, Molecular Docking, and Dynamics Simulations
Source: Foods. 2025 Jun 23;14(13):2202. doi: 10.3390/foods14132202 (PMC12248981; doi:10.3390/foods14132202)
Supplement: Supplementary file 1 [file foods-14-02202-s001.zip › foods-3693758-supplementary.pdf]

Supplementary Materials

Table S1 *Ulva prolifera* protein selected from the NCBI database

Ulva prolifera protein	Accession Number	Amino acid number
pyruvate orthophosphate dikinase	AFC75637	899
pyruvate phosphate dikinase 2	WDE94729	972
sedoheptulose-1,7-bisphosphatase	WDE94727	385
ribulose-1,5-bisphosphate carboxylase/oxygenase large subunit (chloroplast)	UEN67742	474
photosystem I assembly protein Ycf4 (chloroplast)	WFS79909	180
photosystem II reaction center protein Ycf12 (chloroplast)	YP_009440079	34
elongation factor Tu (chloroplast)	YP_009440072	407
30S ribosomal protein S9 (chloroplast)	YP_009440061	133
ribosomal protein S7 (chloroplast)	UEN67764	156
ribosomal protein S3 (chloroplast)	UEN67762	231
RNA polymerase beta" subunit (chloroplast)	UEN67756	2751
photosystem II CP47 chlorophyll apoprotein (chloroplast)	UEN67729	508
photosystem I P700 apoprotein A1 (chloroplast)	UEN67723	751
cytochrome f (chloroplast)	UEN67719	309
ATP-dependent zinc metalloprotease FtsH (chloroplast)	YP_009440053	1992
ATP synthase CF1 alpha subunit (chloroplast)	UEN67707	309
phosphoenolpyruvate carboxykinase	WDE94730.1	586
phytoene dehydrogenase	UDM59634	533
DNA-directed RNA polymberase subunit alpha (chloroplast)	YP_009440086	536
photosystem I P700 chlorophyll a apoprotein A1 (chloroplast)	YP_009440066	751
acetyl-coenzyme A carboxylase carboxyl transferase subunit beta (chloroplast)	YP_009440082	312
Phosphoribulokinase	WDE94728	383
chloroplast ribulose-1,5-bisphosphate carboxylase/oxygenase small subunit	WDE94726	180
adenine phosphoribosyltransferase	BCU09066	174
hypothetical protein RF1 (chloroplast)	UEN67769	810
30S ribosomal protein S12 (chloroplast)	UEN67767	123
ribosomal protein S8 (chloroplast)	UEN67765	131
cyctochrome c biogenesis protein (chloroplast)	YP_009440068	381
RNA polymerase alpha subunit (chloroplast)	UEN67753	536

Ulva prolifera **protein**	**Accession Number**	**Amino acid number**
photosystem I P700 chlorophyll a apoprotein A1 (chloroplast)	YP_009440066	751
photosystem II protein D2 (chloroplast)	UEN67731	352
DNA-directed RNA polymberase subunit beta (chloroplast)	YP_009440065	2757
photosystem I assembly protein Ycf4 (chloroplast)	UEN67772	180
ribosomal protein S2 (chloroplast)	UEN67761	237
photosystem I P700 apoprotein A2 (chloroplast)	UEN67724	735
photosystem II protein D1 (chloroplast)	UEN67728	353
LHY	UDM59635	868
plastid 1-deoxy-D-xylulose 5-phosphate synthase plastid	QBP34359.1	713
4-hydroxy-3-methylbut-2-en-1-yldiphosphate synthase precursor	QBP34357	719
